# Encoder-Weighted W-Net for Unsupervised Segmentation of Cervix Region in Colposcopy Images

**DOI:** 10.3390/cancers14143400

**Published:** 2022-07-13

**Authors:** Jinhee Park, Hyunmo Yang, Hyun-Jin Roh, Woonggyu Jung, Gil-Jin Jang

**Affiliations:** 1 School of Electronic and Electrical Engineering, Kyungpook National University, Daegu 41566, Korea; pjhdrm@knu.ac.kr; 2 Neopons, Daegu 41404, Korea; 3 Department of Biomedical Engineering, Ulsan National Institute of Science and Technology, Ulsan 44919, Korea; hmyang@unist.ac.kr (H.Y.); wgjung@unist.ac.kr (W.J.); 4 Department of Obstetrics and Gynaecology, University of Ulsan College of Medicine, Ulsan University Hospital, Ulsan 44033, Korea; 0729345@uuh.ulsan.kr; 5 School of Electronics Engineering, Kyungpook National University, Daegu 41566, Korea

**Keywords:** unsupervised learning, unsupervised segmentation, colposcopy, cervical cancer screening, W-Net

## Abstract

**Simple Summary:**

The cervix region segmentation significantly affects the accuracy of diagnosis when analyzing colposcopy. Detecting the cervix region requires manual, intensive, and time-consuming labor from a trained gynecologist. In this paper, we propose a deep learning-based automatic cervix region segmentation method that enables the extraction of the region of interest from colposcopy images in an unsupervised manner. The segmentation performance with a Dice coefficient improved from 0.612 to 0.710 by applying the proposed loss function and encoder-weighted learning scheme and showed the best performance among all the compared methods. The automatically detected cervix region can improve the performance of image-based interpretation and diagnosis by suggesting where the clinicians should focus during colposcopy analysis.

**Abstract:**

Cervical cancer can be prevented and treated better if it is diagnosed early. Colposcopy, a way of clinically looking at the cervix region, is an efficient method for cervical cancer screening and its early detection. The cervix region segmentation significantly affects the performance of computer-aided diagnostics using a colposcopy, particularly cervical intraepithelial neoplasia (CIN) classification. However, there are few studies of cervix segmentation in colposcopy, and no studies of fully unsupervised cervix region detection without image pre- and post-processing. In this study, we propose a deep learning-based unsupervised method to identify cervix regions without pre- and post-processing. A new loss function and a novel scheduling scheme for the baseline W-Net are proposed for fully unsupervised cervix region segmentation in colposcopy. The experimental results showed that the proposed method achieved the best performance in the cervix segmentation with a Dice coefficient of 0.71 with less computational cost. The proposed method produced cervix segmentation masks with more reduction in outliers and can be applied before CIN detection or other diagnoses to improve diagnostic performance. Our results demonstrate that the proposed method not only assists medical specialists in diagnosis in practical situations but also shows the potential of an unsupervised segmentation approach in colposcopy.

## 1. Introduction

Cervical cancer, the fourth most common cancer in women, is strongly associated with human papillomavirus (HPV) infection [1]. Because cervical cancer can be cured if diagnosed early and treated quickly, its incidence and mortality rates have decreased over the past few decades [2]. Most cervical cancer cases and deaths currently occur in low- and middle-income countries (LMICs), due to a shortage of experienced clinicians, medical facilities for screening, and supplies such as preventive vaccines [3].

Papanicolaou (Pap) smears and colposcopy are generally used to screen for cervical cancer. A potentially precancerous transformation of cervical cells is called cervical dysplasia. The Pap smear is a test that identifies the presence of abnormal cells by examining cells under a microscope. Colposcopy is a procedure that looks for cancerous or abnormal cells by looking at the cervix region through a special magnifying device called a colposcope, with a green filter often used to see blood vessels more clearly. Colposcopy has generally been found to have higher sensitivity and specificity than Pap smears [4,5,6]. If abnormal cells are found during colposcopy, a clinician collects a small amount of tissue for biopsy. However, due to the subjective nature of colposcopy, its accuracy is highly dependent on the clinician’s experience and capacity. Therefore, there is a limit to the use of colposcopy as a screening method, especially in LMICs, due to the lack of specialists experienced in colposcopy.

Deep learning has received significant attention due to its ability to automatically learn and extract meaningful features from input data [7] and has been used to improve performance in various medical fields [8,9]. Deep learning also shows considerable potential in computer-aided diagnosis (CAD). For these reasons, some studies have utilized deep learning methods to assist clinicians in colposcopy. Most studies have focused on cervical intraepithelial neoplasia (CIN) classification [10,11,12,13,14,15,16,17,18], and almost all studies include a pre-processing step to first remove non-cervix regions such as the speculum and vaginal walls, in order to extract only the cervix region, which is the region of interest (ROI) in colposcopy. The performance of cervix segmentation significantly affects the accuracy of diagnosis when analyzing colposcopy and is an essential step in training deep learning-based models. Previous studies utilized segmentation and object detection models to extract the ROI in a supervised manner [19,20]. Supervised learning can achieve high performance, but annotating the cervix region for every colposcopy image is not only a subjective judgment but is also burdensome for doctors. For these reasons, some studies have attempted to extract the ROI without annotation masks, in an unsupervised manner, based on hand-crafted features, such as geometrical curvature characteristics or color information [21,22,23]. A study [24] has attempted unsupervised cervical ROI segmentation consisting of complex steps in the consideration of the characteristics of colposcopy but still requires image pre-processing.

In this paper, we introduce a fully unsupervised method for the cervix region segmentation in colposcopy using an adaptation of W-Net [25], a deep learning model for unsupervised image segmentation. We propose to substitute the graphcut-based loss with a combination of cross-entropy and total-variation loss, reducing the computational overhead and time for model training. To improve the cervical ROI segmentation performance, an encoder-weighted learning approach in shallow network architecture is proposed. The proposed method improves the segmentation performance with a Dice coefficient from 0.6120 to 0.7100 compared to baseline W-Net and produces enhanced segmentation masks without pre- and post-processing. In addition, the training time required for optimal performance is reduced from 10.6 h to 3.4 h. Experimental results demonstrate the potential of an end-to-end unsupervised approach in colposcopy segmentation. In summary, the contributions of this study are as follows:

- An end-to-end unsupervised method is proposed to efficiently segment cervical ROI using the adaptation of W-Net in colposcopy. To the best of our knowledge, this is the first study to resolve fully unsupervised cervix region segmentation without pre- and post-processing.
- CT-loss (a combination of cross-entropy loss and total-variation loss) is proposed to reduce the computational overhead and time for model training compared to baseline W-Net, and experimental results confirmed that the proposed method reduced the training time from 10.6 h to 4 h while improving the segmentation performance with a Dice coefficient from 0.6120 to 0.6870.
- An encoder-weighted learning approach in shallow network architecture is proposed to improve the cervical ROI segmentation performance. Experimental results showed the best segmentation performance with a Dice coefficient of 0.7100.

## 2. Materials and Methods

### 2.1. Datasets

In this study, we use the Intel and MobileODT Cervical Cancer screening database (https://www.kaggle.com/competitions/intel-mobileodt-cervical-cancer-screening accessed on 8 March 2021). There are three different cervix types (types 1, 2, and 3), and the purpose of this challenge is to develop an accurate algorithm for identifying cervix types using colposcopy images. For experiments, we select type 1 data consisting of about 1500 images. Since the original data contain many duplicates and low-quality images, such as being excessively shaky and out-of-focus, these poor-quality images were excluded. Excessively enlarged images where all regions were cervix regions, and green-filtered images, were excluded as well. Consequently, the proposed model is trained and evaluated using approximately 270 colposcopy images, including before and after aceto-whitening and after iodine staining. For evaluation, the authors annotate the cervix region as an ROI of colposcopy, and these annotations are confirmed by an experienced gynecologist. Annotations are never used for model training but only for evaluating the performance of cervix region segmentation.

### 2.2. Deep Learning Model for Unsupervised Segmentation

We discuss two studies related to our method, involving novel convolutional neural networks (CNN)-based approaches to unsupervised image segmentation. 

#### 2.2.1. Baseline W-Net

W-Net [25] is implemented by concatenating two U-Net [26] architectures into a single autoencoder [27]. The U-Net is a U-shaped network for biomedical image segmentation. An autoencoder, which consists of an encoder and a decoder network, is a specific type of neural network with the same input and output. The encoder maps the input to a smaller-sized set of codes, and the decoder reconstructs the input from the codes. Through iterative model training, the autoencoder learns to compress unlabeled data into more efficient code sets. The W-Net encoder can yield an abstractive code set of the raw input image by employing autoencoder architecture, and the coded values are segmented into *K* classes before model training using a graph-based normalized cut method [28], called soft N-cut loss. The W-Net decoder is trained to map the segmented image that is the output of the W-Net encoder to the input image, by minimizing the reconstruction error between the input image and the predicted output of the W-Net decoder. During training, soft N-cut loss and reconstruction error are jointly minimized, and the encoded image is then post-processed to generate the final segmentation result. One study [29] adopted W-Net for the segmentation of confocal scanning laser ophthalmoscopy (cSLO) images. The main difference between the W-Net proposed in [29] and the baseline W-Net is that a pooling layer is added before calculating the soft N-cut loss, to reduce memory consumption.

#### 2.2.2. CNN-Based Method

Kim et al. [30] proposed a CNN-based differentiable feature clustering method that jointly optimizes the pixel labels and feature representations and produces segmentation masks through iterative model training. First, a normalized response map is obtained by passing the image through feature extraction and then response map normalization components. This response map implies the probability that each pixel belongs to each of the *K* classes, where *K* must also be set manually before training the model. Then, a pseudo segmentation mask is obtained by selecting the maximum probability among the *K* classes for each pixel. The network is trained by minimizing the sum of feature similarity loss and spatial continuity loss using response maps and pseudo segmentation masks. The feature similarity serves to assign the same label to pixels with similar characteristics, and the spatial continuity facilitates cluster separation by favoring the assignment of cluster labels to be the same as those of the adjacent pixels. Through iterative model training, a segmentation result can be produced. We refer to this method as the CNN-based method in the following sections.

### 2.3. Proposed Method

#### 2.3.1. CT-Loss: Cross-Entropy and Total-Variation Loss

We adopted W-Net [25], which is a deep learning model for fully unsupervised image segmentation. It is used to capture the cervix region as an ROI of colposcopy. The main limitations of the baseline W-Net for colposcopy images are (1) the use of memory- and time-consuming graphcut operations to calculate the soft N-cut loss; and (2) post-processing due to over-segmented results from the input image. Because medical images are usually in higher resolution than other types of images, most studies scale or tile the medical images before training CNN models. To calculate the soft N-cut loss, we need to measure the sum of all weights between the input image and the segmented mask for all of the pixels. The larger the image, the higher the computational overhead. As a baseline model of our work, we adopt the W-Net architecture with an additional pooling layer before soft N-cut loss calculation [29]. The pooling layer reduces the output map size and therefore can reduce memory requirement. However, soft N-cut loss requires huge memory and computational overhead.

**Figure 1:**
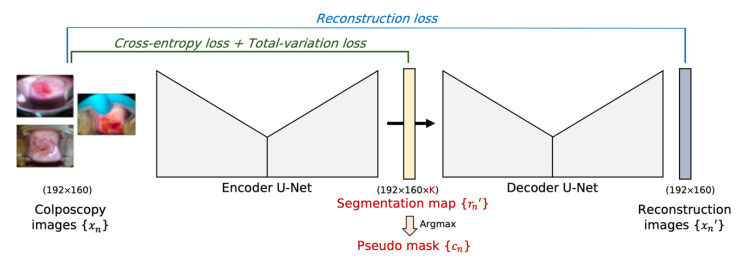
Overview of the proposed W-Net with CT-loss.

Figure 1 shows an overview of the proposed W-Net. To overcome the limitations of the baseline W-Net, we replace the soft N-cut loss with a combination of cross-entropy and total-variation loss (CT-loss) inspired by the CNN-based method [30], as follows:

(1) $$L_{enc}=L_{CT}=L_{\;cross-entropy}+L_{\;total-variation},\;L_{\;cross-entropy}=L\left(\left\{r_{n}^{\prime },\;c_{n}\right\}\right)=-\overset{K}{\underset{i}{\sum }}c_{i}\log\left(r_{i}^{\prime }\right),\;L_{\;total-variation}=L\left(\left\{r_{n}^{\text{’}}\right\}\right)=\overset{W-1}{\underset{\xi }{\sum }}\overset{H-1}{\underset{\eta }{\sum }}∥r_{\xi +1,\eta }^{\text{’}}-r_{\xi ,\eta }^{\text{’}}{∥}_{1}+∥r_{\xi ,\eta +1}^{\text{’}}-r_{\xi ,\eta }^{\text{’}}{∥}_{1},$$

where $\left\{r_{n}^{\prime }\right\}$ denotes denote the normalized segmentation map of the sample *n*, and $\left\{c_{n}\right\}$ is the pseudo segmentation mask obtained by the index maximizing the value of the normalized segmentation map $\left\{r_{n}^{\prime }\right\}$ . W and H represent the width and height of an input image, and $r_{\xi ,\eta }^{\prime }$ represents the pixel value at ( ξ, η) in the normalized segmentation map $\left\{r_{n}^{\prime }\right\}$ . This CT-loss $L_{CT}$ reduces memory usage and training time. Moreover, due to the nature of the total-variation loss, the segmentation mask can be more condensed than the baseline method without post-processing. Each pixel in the segmentation map has the probability of belonging to each of the *K* classes, and the number of classes *K* should be determined manually. The pseudo segmentation mask can be obtained by choosing the maximum probability from among the *K* classes. The average probability of each pseudo segmentation mask is considered as the confidence score of each image, and this confidence score was used as the criterion for CNN training.

#### 2.3.2. EW Learning: Encoder-Weighted Learning for W-Net

For the proposed W-Net, the initial pseudo segmentation mask affects the segmentation performance. In other words, the initial pseudo segmentation mask is important for model convergence. The training procedure for the baseline W-Net is to update the encoder U-Net first and then update the entire W-Net (both the encoder U-Net and decoder U-Net). In this procedure, the pseudo segmentation mask is initially changed a little at every iteration, so if the initial value is not appropriate, the model will not be optimized.

To reduce the dependency on the initial pseudo mask, encoder-weighted (EW) learning is proposed. The encoder U-Net is first trained using $L_{enc}$ during i epochs, which is the number of epochs to train the encoder U-Net. Once the encoder U-Net becomes stable to some extent, the entire W-Net is trained using $L_{entire}$ as follows:

(2) $$L_{entire}=L_{\;reconstruction}=L_{\;MSE}=\frac{1}{N}\overset{N}{\underset{i}{\sum }}\left(x_{i},\;x_{i}^{\prime }\right).$$

**Figure 2:**
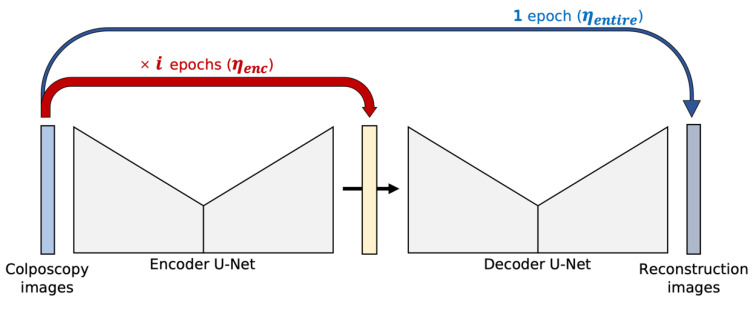
Proposed encoder-weighted learning scheme.

Figure 2 illustrates the proposed learning scheme. The encoder U-Net learning step is repeated i epochs with a learning rate $\eta_{enc}$ . Then, a single learning step of the entire encoder and decoder U-Nets is performed. The different learning rate $\eta_{entire}$ is used for the decoder U-Net. With EW learning, the encoder U-Net trains more epochs than the decoder U-Net, but balanced learning of the encoder and decoder is important. Therefore, we set relatively small learning later for encoder U-Net $\left(\eta_{enc}\right)$ than for decoder U-Net $\left(\eta_{entire}\right)$ to avoid bias to the encoder U-Net.

The proposed EW learning increases the model training time due to pre-training on the encoder U-Net. To efficiently apply the proposed EW scheme, we decrease the model depth of the baseline W-Net architecture. The number of convolutional filters in each layer for the encoder and decoder U-Net of the baseline W-Net architecture is (64, 128, 256, and 512) with a depth of 4. In the proposed shallow architecture for encoder weighted learning, the number of convolutional filters in each layer is (64, 128, and 256) with a depth of 3.

## 3. Results

### 3.1. Implementation Details

All images were resized and normalized so that the pixel values were in the range (0, 1) for efficient CNN training. Various data-augmentation techniques such as flipping and blurring were used to augment the training dataset for model training. We set K = 2, the number of segmentation classes, and set *i* = 7, the number of epochs for training encoder U-Net. The hyperparameters for model training were chosen using early stopping, namely, we selected the epoch that gave the best average confidence score for all inputs. We utilized an NVIDIA RTX 2080 Ti GPU with CUDA 11.0 and cuDNN 8.0.5 for our experiments. We implemented these networks using PyTorch [31] following the descriptions found in each paper and their GitHub repositories. To train the proposed W-Net, we used a combination of cross-entropy and total-variation loss (CT-loss) for the encoder and mean squared error (MSE) loss for the reconstruction error for the entire W-Net (both encoder and decoder). We also used the Adam optimizer with a learning rate of 7 × 10^−5^ for the encoder and 1 × 10^−3^ for the decoder and set the maximum number of epochs to 500.

### 3.2. Qualitative and Quantitative Results

The segmentation performance was evaluated by the Dice similarity coefficient [32], which measures the similarity between the two samples.

(3) $$Dice\;coefficient=\frac{2\left|X\cap^{\;}Y\right|}{\left|X\right|+\left|Y\right|}=\frac{2*TP}{\left(TP+FP\right)+\left(TP+FN\right)}.$$

Since the model size of the CNN-based method depends on the number of convolutional components (*M*) for feature extraction, we adjusted the *M* value for a fair comparison with the proposed shallow method, so that both models had a similar number of trainable parameters.

We compared the number of trainable parameters, the training time, and the average Dice coefficient for the evaluation sets (Table 1). The substitution of graphcut soft N-cut loss with a combination of cross-entropy loss and total-variation loss (CT-loss) reduced the training time from 10.6 h to 4 h with the same number of trainable parameters. It also improved the Dice coefficient from 0.6120 to 0.6870. The depth of the network architecture, and the number of convolutional layers, affect the training time more than the number of convolutional filters. The depth of the CNN-based network is deeper than that of the W-Net architecture because it consists of iterative components with convolutional layers. Therefore, although the number of trainable parameters for the CNN-based method (3.55 M) is smaller than the baseline W-Net (12.3 M), it takes relatively longer to train. The proposed method with CT-loss also achieves a slightly better Dice coefficient than the CNN-based method. The proposed method 2 (CT-loss and EW learning in a shallow architecture) achieves the best cervical ROI segmentation performance of 0.7100 (10% better than the baseline W-Net) with a reduced model optimization time compared to the comparative method. These results demonstrate the effectiveness of CT-loss and EW learning in the shallow architecture of the proposed method.

The Wilcoxon signed-rank test [33] was performed to determine whether pairwise performance differences are statistically significant with each other. Each *p*-value is computed using the Dice coefficient between the cervical ROI segmentation mask and annotation for the individual samples. The *p*-values for each null hypothesis are tabulated in Table 2. The performance between the proposed method (both Proposed 1 and Proposed 2) and the baseline W-Net (Graphcut W-Net) shows a statistically significant difference. The proposed method 2 also shows a statistically significant performance improvement over the CNN-based method. However, as shown in Table 1, the Dice coefficient of the proposed 1 is slightly better than the CNN-based method, and there is no statistically significant difference between the two methods as a result of the significance test.

**Figure 3:**
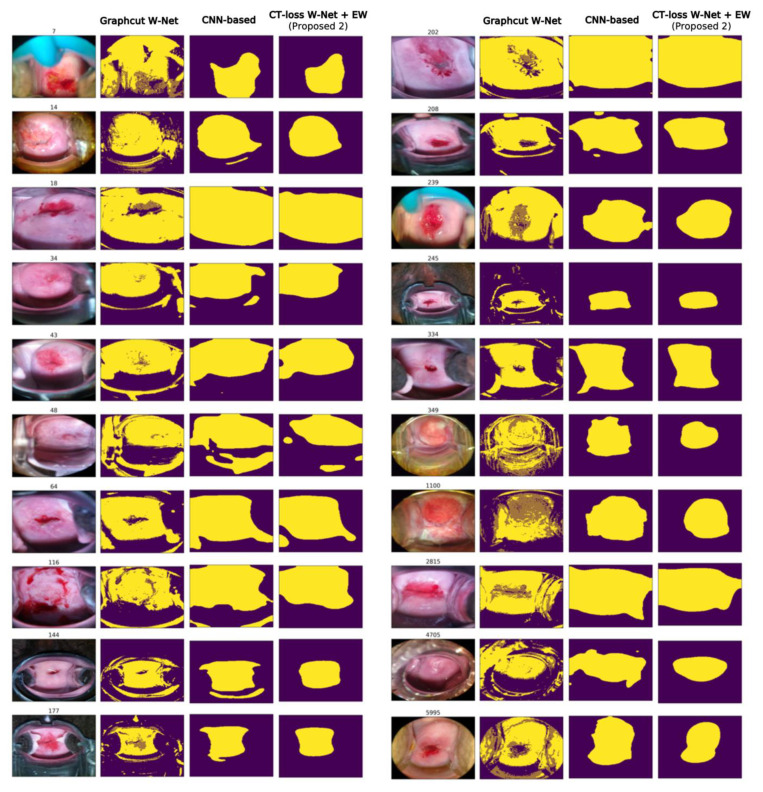
Examples of cervical ROI segmentation comparison.

The cervical ROI segmentation masks for each comparison method can be seen in Figure 3. The baseline graphcut W-Net can capture colposcopy ROI well, and our method shows a similar tendency to the baseline W-Net. However, the proposed method, CT-loss W-Net with EW learning, yields more condensed masks without post-processing than the baseline W-Net. As can be seen in Figure 3, the proposed method (CT-loss + EW learning) has less tendency to include non-ROI regions and focuses more on the cervix region compared to the CNN-based method (e.g., sample index: 15, 48, 116, and 144).

### 3.3. Ablation Study

Ablation studies were performed by varying the number of epochs, i , of the encoder U-Net for EW learning. If i=1 , it is the same as the baseline W-Net training procedure: update the encoder U-Net first, then update the entire W-Net. A large i allows the model to focus on the encoder U-Net, allowing the encoder to produce a more consistent segmentation mask. However, the decoder U-Net training is also an important component, and too many epochs to encoder U-Net can break the entire W-Net training balance.

We tested the effect of EW learning using 4 different i (3, 6, 7, and 10) and compared the Dice coefficient on the evaluation sets (Table 2). The segmentation performance on the evaluation sets improved as i increased to 3 and 6. The optimal epoch with the best performance was i=7 , and when i=10 , the performance was rather decreased. The Wilcoxon signed-rank test is also performed to validate the significance of the performance improvement between different i . As shown in Table 3, there is a statistically significant improvement in cervix segmentation performance when i=7 compared to other i . These results indicate that the proposed encoder-weighted learning can achieve performance improvement by controlling the excessive change of the initial pseudo mask.

## 4. Discussion

Colposcopy analysis usually consists of three steps: the detection of the cervix region, the extraction of important features such as the transformation zone and the abnormal vessel, and image-based interpretation and diagnosis. Our study focuses on the first step, the detection of the cervix region. The purpose of the first step is to remove non-cervix regions, allowing the clinician to focus on the important lesion and to help extract the important features needed for diagnosis. Consequently, the detection of the cervix region can improve the performance of image-based interpretation and diagnosis. In other words, the cervix segmentation affects the accuracy of diagnosis when analyzing colposcopy.

In this study, we introduce the fully unsupervised cervix region segmentation of colposcopy using an adaptation of W-Net. The accuracy of colposcopy is highly dependent on the clinician’s competence, and the segmentation of the cervix region is important for the accuracy of the diagnosis. Most previous studies utilized segmentation and object detection models to extract the ROI in a supervised manner, which led to a burdensome and time-consuming annotation task for gynecologists. In addition, finding an appropriate post-processing method is also subjective and is generally achieved using heuristics. It is desirable to minimize post-processing as this strongly depends on the input characteristics and the purpose of the task. The proposed method can segment the ROI of colposcopy in an unsupervised manner, and no pre- and post-processing are required.

Although the proposed method produces a reasonable cervical ROI segmentation mask, for some samples the predicted ROI representing the cervix region contains non-cervix regions, such as the speculum, vaginal wall, or glaring area. Nevertheless, our segmentation results could suggest a general ROI in colposcopy. In addition, our method could produce a cervix segmentation mask with better quality than the comparative methods while reducing the computational overhead and time for model training. The proposed method can assist the diagnosis by suggesting where the clinicians should focus when analyzing colposcopy images. It can be used as a pre-processing method for automatic CIN detection or other diagnoses to improve diagnostic performance. Moreover, the experimental results demonstrate the potential of an unsupervised segmentation approach in colposcopy. To the best of our knowledge, this is the first study to resolve fully unsupervised cervix region segmentation end-to-end without any pre- and post-processing.

## 5. Conclusions

We propose an adaptation of W-Net, an end-to-end deep learning model for unsupervised cervix segmentation. To reduce the computational overhead and time required for model training, we propose the loss substitution with CT-loss (a combination of cross-entropy and total-variation loss) in a graphcut loss function. We also propose an encoder-weighted (EW) learning in shallow network architecture to improve cervix segmentation performance. The proposed method was validated on the Kaggle dataset, and the experimental results show that our method can efficiently obtain consistent segmentation masks for the ROI of colposcopy even with a small training dataset. The segmentation performance with a Dice coefficient improved from 0.6120 to 0.7100 by applying the proposed CT-loss and EW learning compared to the baseline graphcut W-Net and CNN-based method. In addition, the time required to train the optimized model was reduced from 10.6 h to 3.4 h.

## Figures and Tables

**Table 1:** Performance comparison results of cervical ROI segmentation.

	Graphcut W-Net [29]	CT-Loss W-Net (Proposed 1)	CNN-Based [30]	CT-Loss W-Net + EW (Proposed 2)
Number of parameters	12.3 M	12.3 M	3.55 M	3.27 M
Training time	10.6 h	4 h	4.4 h	3.4 h
Dice coefficient	0.6120	0.6870	0.6789	0.7100

**Table 2:** Result of significance test between the different methods.

Compared Methods	*p*-Value
Graphcut W-Net vs. Proposed 1	9.36 × 10^−21^
CNN-based vs. Proposed 1	0.86
Graphcut W-Net vs. Proposed 2	4.91 × 10^−20^
CNN-based vs. Proposed 2	5.60 × 10^−6^

**Table 3:** Performance comparison for different i in encoder-weighted learning.

	CT-Loss W-Net + EW			
	EW ( i = 3)	EW ( i = 6)	EW ( i = 7)	EW ( i = 10)
Dice coefficient	0.6591	0.6823	0.7100	0.6908
*p*-value (vs. EW( i = 7))	4.28 × 10^−14^	3.08 × 10^−8^	-	1.00 × 10^−4^

## Data Availability

Data was obtained from https://www.kaggle.com/competitions/intel-mobileodt-cervical-cancer-screening/ (accessed on 8 March 2021) and are available with the permission of after competition entry.
